# Editorial: Genetics of non-syndromic hearing loss

**DOI:** 10.3389/fgene.2025.1665942

**Published:** 2025-08-12

**Authors:** Caio Robledo D’ Angioli Costa Quaio, Saba Battelino

**Affiliations:** ^1^ Hospital Israelita Albert Einstein, São Paulo, Brazil; ^2^ Instituto da Criança (Children’s Hospital), Hospital das Clínicas HCFMUSP, Faculdade de Medicina, Universidade de São Paulo, São Paulo, Brazil; ^3^ Department of Otorhinolaryngology and Cervicofacial Surgery, University Medical Centre, Ljubljana, Slovenia; ^4^ Faculty of Medicine, University of Ljubljana, Ljubljana, Slovenia

**Keywords:** hearing loss, non-syndromic hearing loss, genomics, genetics, rare disease, genetic variants

Non-syndromic hearing loss (NSHL) remains the most common sensory disorder, affecting individuals across the lifespan, from approximately one in every 500 newborns to more than two-thirds of octogenarians (Yamasoba et al., 2013; Shearer and Smith, 2015). Because the condition presents without additional clinical features, gene discovery and molecular diagnosis are essential for counselling families, guiding rehabilitation strategies, paving the way for precision therapeutics, and informing public health policies. With more than 120 genes already implicated and new loci reported each year (Shearer et al., 2017; Shearer and Smith, 2015), the field now faces two intertwined challenges: (a) resolving the extraordinary genetic heterogeneity of NSHL across global populations, and (b) translating that knowledge into equitable, high-yield diagnostic workflows.

The four articles gathered in this Research Topic take complementary approaches to those challenges and, taken together, expand the catalog of pathogenic variants and outline an up-to-date roadmap for the next phase of NSHL genetics.

Studying a socio-economically and ethnically diverse Brazilian cohort, Antunes et al. examined 90 probands with presumed autosomal-recessive NSHL. Using two complementary NGS strategies (a 99-gene hearing-loss panel and whole-exome sequencing) they identified pathogenic variants in 32 of 90 probands (36.7%), uncovering 39 alleles across 24 genes, including 10 previously unreported variants. Notably, causative variants in *SIX1* and *P2RX2* revealed autosomal-dominant inheritance in two families that pedigree analysis had suggested were recessive, and several probands carried variants in genes classically linked to syndromic deafness (e.g., *USH2A*), underscoring the need to differentiate isolated from syndromic presentations because the latter may mandate extra-auditory surveillance. As one of the largest NGS surveys of hearing loss in Latin America, the study confirms the extreme locus heterogeneity of NSHL and demonstrates that multi-gene NGS testing is a highly effective first-tier diagnostic strategy.

Ali et al. applied whole-exome sequencing to 11 Emirati families, of which 45.5% were consanguineous. A molecular diagnosis was achieved in 6 probands (54.5%), identifying pathogenic or likely pathogenic variants in *MYO15A*, *SLC26A4*, and *GJB2*; one nonsense variant in *MYO15A* (p.Tyr1962Ter) was previously unreported. In the remaining five families, the authors detected six variants of uncertain significance in *CDH23*, *COL11A1*, *ADGRV1*, *NLRP3*, and *GDF6* and, through an extensive *in silico* pipeline, classified them as potentially deleterious. The study demonstrates that robust genetic diagnosis is feasible in populations with high consanguinity and underscores the role of molecular results in guiding prognosis, therapy, and reproductive counselling, including pre-conception carrier testing and embryo selection.

Studying a cohort of 152 Chinese families with heterogeneous clinical presentations, Zeng et al. implemented a four-tier stepwise strategy that combined multiplex PCR + high-throughput sequencing, Sanger sequencing, MLPA of the *STRC* gene, and reflex exome sequencing. This pipeline yielded a high diagnostic rate of 73% (111/152). Notably, exome sequencing contributed an additional 18.4% of solved cases and uncovered 21 novel variants in 15 deafness genes. It also re-classified 11 probands as having syndromic hearing loss, a distinction that triggers surveillance of extra-auditory organs and systems. The authors emphasize the value of tailoring test menus to local allele spectra, laboratory infrastructure, and cost constraints. They further observe that although precision therapies remain scarce, advances in inner-ear gene delivery suggest that gene therapy may become a viable option for selected molecular subtypes.

The traditional focus on autosomes has left potential sex-linked contributions underexplored. Naderi et al. investigated X-chromosome variants associated with age-related hearing loss using data from the United Kingdom Biobank, encompassing over 460,000 individuals of White European ancestry. To date, the genetic basis of this kind of hearing loss remains incompletely understood, and X-linked contributions have received limited attention. Through genome-wide association analysis, the authors identified significant associations between age-related hearing loss and variants near *ZNF185*, *MAP7D2*, and *LOC101928437* on the X chromosome. They concluded that X-linked variants account for only a small proportion of the genetic variance in this kind of hearing loss, suggesting a limited role for the X chromosome in this condition. The authors emphasize that replication of these findings in populations with different genetic backgrounds will be essential to validate the observed associations and to determine their broader relevance.

In conclusion, the studies in this Research Topic not only deepen our understanding of the genes and variants that shape the auditory landscape but also chart practical paths toward precision medicine for all individuals living with NSHL, including (a) awareness of their molecular diagnosis, which has significant psychological, familial, and social implications; (b) individualized surveillance and management, particularly for those with syndromic forms of hearing loss that require specialized, multisystem care; (c) informed family planning; and (d) access to assisted‐reproduction options such as pre-implantation genetic testing. Nevertheless, a substantial proportion of cases remains genetically unresolved, underscoring the need for novel approaches to close the diagnostic gap.

The study of oligogenic and multifactorial variation, X-linked modifiers, and non-coding regulatory regions of the genome are among the approaches needed to move beyond a strictly Mendelian script. Multi-omics strategies, such as long-read sequencing to resolve structural variants and single-cell epigenomics to map cochlear regulatory landscapes, are poised to uncover additional unsolved forms of NSHL.

Looking ahead, two areas warrant particular attention. First, functional validation of variants of uncertain significance: scalable inner-ear organoid and animal-model platforms will be essential to convert variant catalogs into mechanistic insight and therapeutic targets. Second, the development of scalable frameworks that harness molecular diagnoses to match each patient with emerging precision treatments, such as antisense oligonucleotides, and gene replacement and genome editing, thereby transforming genetic knowledge into personalized therapy.

We thank the authors for their high-quality contributions and the reviewers for their expert guidance. We trust these papers will spark further collaborations that bridge basic discovery and clinical application. Our ultimate goal is that every person with hearing loss can benefit from an early, accurate molecular diagnosis and tailored care.
